# Effects of deep brain stimulation on non motor fluctuations in Parkinson’s disease (assessed with the NMF severity scale)

**DOI:** 10.1016/j.prdoa.2026.100426

**Published:** 2026-02-08

**Authors:** Christian Matta, Camille Comet, Corentin Gaillard, Florent Faggianelli, Stephan Grimaldi, Tatiana Witjas, Solène Ansquer, Cécile Hubsch, Mathieu Anheim, Caroline Moreau, Elodie Hainque, Sophie Drapier, Béchir Jarraya, Chloé Laurencin, Dominique Guehl, Lucie Hopes, Christine Brefel-Courbon, Melissa Tir, Ana Marques, Tiphaine Rouaud, David Maltete, Caroline Giordana, Karine Baumstarck, Olivier Rascol, Jean-Christophe Corvol, Fabienne Ory-Magne, Anne-Sophie Rolland, David Devos, J-P Azulay, Alexandre Eusebio, PREDISTIM Study Group

**Affiliations:** a Department of Neurology, Saint Joseph University, Beirut, Lebanon; b Department of Neurology, University Hospital Timone, Marseille, France; c Centre Hospitalier Universitaire de Poitiers, France – Centre d’investigation clinique CIC INSERM 1402, France; d Service of Neurology, Department of Clinical Neurosciences, Lausanne University Hospital (CHUV) and University of Lausanne (UNIL), Lausanne, Switzerland; e French Clinical Research Network for Parkinson’s Disease and Movement Disorders (NS-PARK), France; f Department of Neurology, Parkinson Expert Centre, U964/UMR7104, France; g Strasbourg University Hospital, University of Strasbourg, INSERM, CNRS, Strasbourg, France; h Strasbourg Institute of Neuroscience, Strasbourg, France; i DD and ASR Department of Medical Pharmacology, Neurology and Movement Disorders Department, Referent Center of Parkinson’s Disease, CHU of Lille, Univ. Lille Neuroscience & Cognition, Inserm, UMR-S1172, NS-Park Network France, Lille, France; j Département de Neurologie, APHP, Paris, France; k Neurologie, CHU Pontchaillou, Rennes, France; l Neuroscience, Hospital Foch, Suresnes, Île-de-France, France; m CNRS UMR 5229, Bron, France; n Centre Expert Parkinson, Bron, France; o Pôle de Neurosciences Cliniques, CHU de Bordeaux, Bordeaux, France; p CNRS UMR 5293, Institut des Maladies Neurodégénératives, Bordeaux, France; q Neurology, Centre Hospitalier Universitaire de Nancy, Nancy, Lorraine, France; r Department of Clinical Pharmacology and Neurosciences, Parkinson Expert Centre, Centre d’Investigation Clinique CIC1436, University Hospital of Toulouse, NeuroToul COEN Centre Inserm UMR-1214, NS-PARK Network, Toulouse, France; s Neurology, CHU Amiens-Picardie, Amiens, Hauts-de-France, France; t Department of Neurology, Clermont-Ferrand University Hospital, Université Clermont Auvergne, Clermont-Ferrand, France; u Neurology, University Hospital Centre Clermont-Ferrand, Clermont-Ferrand, France; v Department of Neurology, Nantes University Hospital, Nantes, France; w Department of Neurology, Rouen University Hospital and University of Rouen, France; x Neurology Department, Centre, Nice, France; y Aix Marseille Université, EA 3279 Self-Perceived Health Assessment Research Unit, Marseille, France; z Department of Neurosciences and Clinical Pharmacology, Parkinson Expert Centre, Centre d’Investigation Clinique CIC1436, NeuroToul COEN Centre, NS-PARK/FCRIN Network, France; aa University Hospital of Toulouse, INSERM, University of Toulouse 3, Toulouse, France; ab Département de Neurologie, CIC Neurosciences, Sorbonne Université, Assistance Publique Hôpitaux de Paris, Paris Brain Institute (ICM), Inserm, CNRS, Hôpital Pitié-Salpêtrière, NS-PARK/FCRIN Network, Paris, France; ac French Clinical Research Network for Parkinson’s Disease and Movement Disorders (NS-PARK), France; ad Departments of Neurosciences, Parkinson Expert Centre, Clinical Investigation Center CIC1436, Toulouse University Hospital, INSERM, University of Toulouse 3, Toulouse, France; ae Department of Medical Pharmacology, Neurology and Movement Disorders Department, Referent Center of Parkinson’s Disease, CHU of Lille, Univ. Lille Neuroscience & Cognition, Inserm, UMR-S1172, Lille, France; af Medical Pharmacology, Lille University Medical Center, Lille, France University of Lille, Inserm, CHU Lille, UMR-S1172 Lille Neuroscience & Cognition (LilNCog), Lille, France; ag Department of Medical Pharmacology, CHU de Lille, France

**Keywords:** Deep brain stimulation, Parkinson’s disease, Non-motor fluctuations, Subthalamic nucleus, Predictive factors of DBS response

## Abstract

**Background:**

Deep Brain Stimulation (DBS) of the subthalamic nucleus (STN) is a well-established treatment for motor fluctuations (MF) in Parkinson’s disease (PD), but its impact on non-motor fluctuations (NMF) remains unclear. As NMFs are frequent, disabling, and affect quality of life, understanding their response to DBS is critical.

**Objectives:**

To assess the presence of NMF after STN-DBS, identify preoperative factors of improvement, and compare NMF responses to DBS and levodopa.

**Methods:**

This project is an ancillary study of the French multicenter PREDISTIM cohort. We used the validated Non-Motor Fluctuation Severity Scale (NMF2S) to evaluate NMFs one year after STN-DBS and before surgery when data were available. Evaluations were performed under standardized conditions (OFF-Dopa/OFF-Stim vs. OFF-Dopa/ON-Stim).

**Results:**

We included 284 PD patients assessed one year after STN-DBS using the NMF2S scale. Preoperative data were available for 153 patients. Evaluations were performed under standardized stimulation conditions (OFF-Dopa/OFF-Stim vs. OFF-Dopa/ON-Stim).

STN-DBS led to a 41.1% reduction in NMF severity, with anxiety, concentration difficulties, and pain showing the most improvement. However, DBS effects were less pronounced than those of levodopa, especially for psychiatric symptoms. NMF improvement did not correlate with motor improvement. Among all preoperative variables, only the levodopa response in the cognitive domain was associated with post-DBS NMF benefit.

**Conclusions:**

STN-DBS significantly improves NMFs, although to a lesser extent than levodopa. The dissociation between motor and non-motor responses underscores the need for specific markers to predict NMF outcomes. These findings support the integration of NMF assessment into DBS indications and patient selection.

## Highlights

• STN-DBS reduces non-motor fluctuations (NMF) by 41.1% at one year.
• Anxiety, pain, and concentration showed most improvement post-DBS.
• DBS was less effective than levodopa for NMF, especially for psychiatric symptoms.
• Motor and non-motor response to DBS were not correlated.
• Only cognitive levodopa response predicted DBS benefit on NMF.

## 1 Introduction

The treatment of Parkinson’s disease (PD) primarily relies on dopaminergic therapy, particularly levodopa, which is effective for both motor and non-motor symptoms. However, with disease progression and long-term treatment, many patients develop motor fluctuations (MF) as well as fluctuations in non-motor symptoms, including psychiatric, cognitive, and autonomic manifestations. These non-motor fluctuations (NMF) are frequent, often disabling, and substantially contribute to impaired quality of life [1].

Previous studies have shown that NMF may occur independently of motor fluctuations or in parallel with them, highlighting the complex interplay between motor and non-motor aspects of PD [1], [2], [3]. In clinical practice, non-motor fluctuations are defined as variations throughout the day, a definition that is inherently influenced by sampling and patient recall and may therefore introduce bias. This methodological limitation complicates the accurate characterization of the acute responsiveness of non-motor symptoms to therapeutic interventions.

Deep brain stimulation (DBS) of the subthalamic nucleus (STN) is an established therapy for advanced PD, with well-documented efficacy on motor fluctuations. Its effects on non-motor symptoms and non-motor fluctuations, however, are less well understood. Studies suggest that DBS may improve some non-motor fluctuations (NMF) [4], [5], but with heterogeneous outcomes and its overall impact and mechanisms remain unclear. Effects vary by symptom and patient, and are not consistently observed [6]. While predictive factors for motor response to DBS are well established [7], little is known about predictors of NMF response.

In order to evaluate the immediate impact of DBS on NMF, in our study, we defined fluctuations as the difference between standardized ON and OFF states using the NMF2S scale. The NMF2S is a clinician-administered, real-time scale that quantifies the severity of 11 fluctuating non-motor symptoms [6]. This approach provides an objective and reproducible measure of non-motor symptom responsiveness, while acknowledging that it does not capture continuous day-long variation.

In this context, the present study aimed to further characterize non-motor fluctuations in PD patients treated with STN-DBS. Specifically, we evaluated the acute effect of DBS on NMF one year after surgery using the NMF2S, compared the non-motor effects of DBS with those of levodopa, and explored clinical and dopaminergic factors associated with postoperative NMF severity and improvement. By addressing these questions in a large multicenter cohort, this study seeks to clarify the magnitude, variability, and potential determinants of non-motor responses to STN-DBS, thereby filling an important gap in the current literature.

## 2 Materials and methods

### 2.1 Study design

#### 2.1.1 Population

This study is ancillary to the multicenter PREDISTIM study (evaluating the predictive factors for the therapeutic response of subthalamic stimulation on Quality of life (QoL) in PD), funded by the French Ministry of Health and France Parkinson, and supported by the NS-Park/FCRIN network. Patients were recruited across 17 French centers.

Eligible participants had undergone bilateral STN-DBS one year earlier, were aged 18–75, met UKPDSBB diagnostic criteria [8], were fluent in French, and able to complete questionnaires. Patients with disease duration < 5 years or who did not meet standard surgical criteria (including dementia, major psychiatric disorders, or MRI abnormalities) were excluded.

A total of 284 patients met the inclusion criteria.

#### 2.1.2 Data collection

The primary objective was to evaluate the effect of DBS on NMF at one year (V2). Predictive analyses were restricted to patients with available preoperative NMF2S data (V1), resulting in fewer patients at V1 than V2.

#### 2.1.3 Assessments

We collected demographic data and key clinical parameters, including motor complications, Hoehn–Yahr stage, Schwab and England scale, MDS-UPDRS, and PDQ-39 [[9], [10]].

To assess NMF, we used the Non-Motor Fluctuations Severity Scale (NMF2S) [6], which quantifies 11 fluctuating non-motor symptoms in OFF and ON conditions, each rated from 0 to 10 (total 0–110). Unlike retrospective scales [[11], [12]], the NMF2S provides an objective, real-time evaluation across three domains (dysautonomic, psychiatric, cognitive). It captures instantaneous symptom severity in standardized ON and OFF states, reducing recall bias, although it does not represent continuous daily variation.

Post-surgical MDS-UPDRS III and NMF2S assessments were performed under OFF-Dopa/OFF-Stim and OFF-Dopa/ON-Stim conditions. “OFF-Dopa” corresponded to overnight withdrawal, “OFF-Stim” to DBS deactivation for at least 1.5 h, and “ON-Stim” to evaluation ≥ 20 min after reactivation.

#### 2.1.4 Ethical considerations

This study is an ancillary investigation of the PREDISTIM study, which was approved by the local institutional review board (CPP Nord Ouest IV, Lille, France; study reference: 2013-A00193-42; registered under NCT02360683 on ClinicalTrials.gov). All participants provided written informed consent before enrollment. The study adhered to the methods, guidelines, and regulations outlined in the approved protocol.

### 2.2 Statistical analysis

Detailed statistical analyses are provided in the Supplementary Materials.

In a few words, the impact of STN-DBS on NMF was evaluated using Wilcoxon signed-rank tests on global and domain-specific NMF2S scores between OFF- and ON-stimulation conditions. Changes in individual symptoms were further examined, and Spearman’s correlations were applied to assess associations with clinical variables and the relationship between motor and non-motor responses. For item-level analyses of the 11 NMF2S items (Table 2), p-values from paired OFF- vs ON-stimulation comparisons were corrected for multiple comparisons using a Bonferroni correction (m = 11; α_Bonferroni = 0.05/11 = 0.0045). Preoperative clinical characteristics and levodopa challenge outcomes were also analyzed for their association with NMF improvement at one year and explored as candidate factors associated with post-DBS NMF changes.

Given the exploratory nature of this ancillary study, the limited number of patients with complete preoperative NMF2S data, and the strong collinearity between candidate clinical variables, multivariable regression analyses were not performed. Instead, we deliberately used univariable and correlational analyses to identify associations, and all results should be interpreted as hypothesis-generating rather than confirmatory.

## 3 Results

### 3.1 Population characteristics

We included 284 patients from the PREDISTIM cohort who had completed the NMF2S questionnaire one year after surgery (V2) (177 men; median age: 59 ± 7 years; median disease duration: 11 ± 4 years).

Among these patients, associations between preoperative variables and post-DBS NMF outcomes were examined in 153 individuals who had also completed the NMF2S questionnaire preoperatively (V1). The number of post-operative patients exceeded that of pre-operative patients, as the primary objective of this study was to assess the impact of DBS on NMF in individuals who had already undergone device implantation. Within this cohort, a subset of patients had completed the NMF2S assessment prior to surgery, although this was not the case for all.

Table 1 summarizes the main patient characteristics at inclusion (V2).

**Table 1:** Main characteristics of the cohort.

**Characteristics V2** 1 year post-surgery		**V1**Before surgery
Subjects (N)	284	153
Gender	62.3% male; 37.7% female	65.4% male; 34.6% female
Age (mean[year], SD)	59.5 (7.3)	59.2 (7.7)
Disease duration (mean[year], SD)	10.95 (4.2)	10.84 (3.8)
PDQ-39 (mean, SD)	44.2 (21.4)	50.2 (20.2)
Hoehn & Yahr OFF (mean, SD)	2.4 (0.8)	2.5 (0.8)
Hoehn & Yahr ON (mean, SD)	1.3 (0.9)	1.2 (0.8)
Schwab & England (OFF) (mean, SD)	70% (20%)	70 ( 18%)
Schwab & England (ON) (mean, SD)	90% (10%)	94% (9%)
MDS UPDRS I (mean, SD)	10.4 (5.8)	11.6 (5.3)
MDS UPDRS II OFF (mean, SD)	14.9 (8.2)	17.6 (7.6)
MDS UPDRS II ON (mean, SD)	7.9 (5.7)	6.4 (5.9)
MDS UPDRS III OFF-Dopa/OFF-Stim (mean, SD)	43.5 (16.0)	41 (15.7)
MDS UPDRS III OFF-Dopa/ON-Stim (mean, SD)	18.2 (12.1)	NA
MDS UPDRS III modification by stimulation (mean, SD)	58.2% (22.6%)	NA
MDS UPDRS IV (mean, SD)	4.7 (3.6)	8.3 (3.5)

PDQ39: Parkinson's Disease Questionnaire. HY: Hoehn & Yahr. SE: Schwab & England. MDS-UPDRS III STIM EFFECT: instantaneous MDS-UPDRS III score after stimulation start-up.

### 3.2 Acute effect of DBS activation (ON/OFF) on NMF

**Fig. 1:**
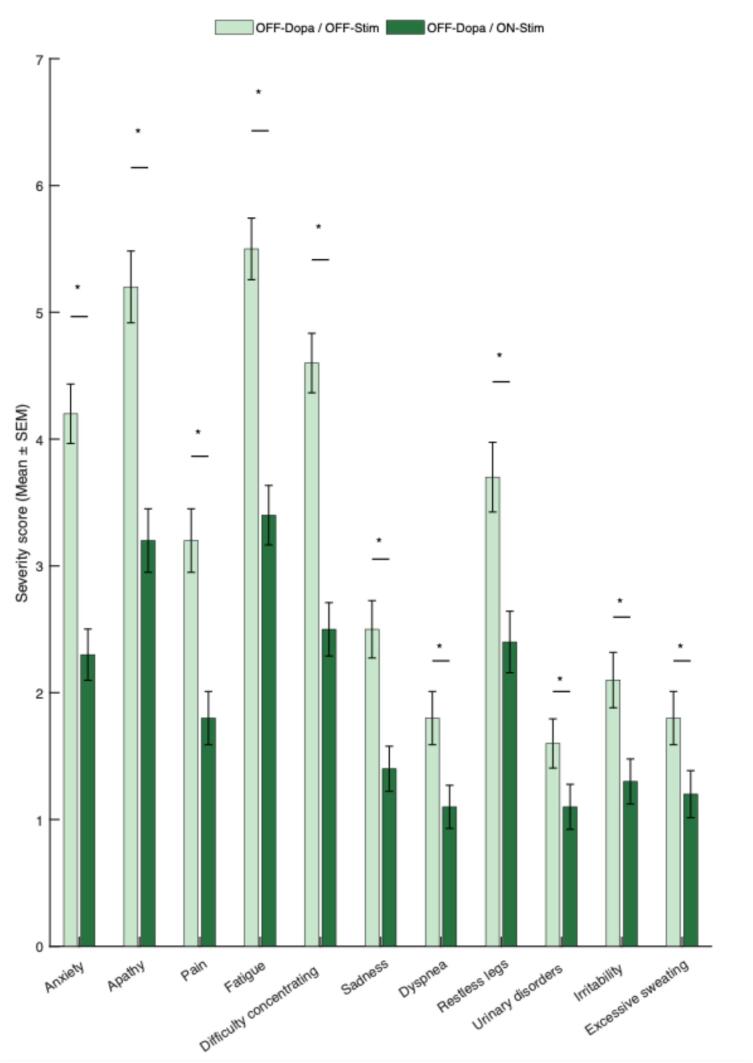
NMF2S scores in the OFF-Dopa/OFF-Stim and OFF-Dopa/ON-Stim conditions.

Data from the NMF2S questionnaire at V2 including the percentage of patients presenting each item, mean NMF2S scores in OFF Stim and ON Stim conditions, mean improvement in scores between OFF and ON conditions, and percentages of patients who improved, remained stable, or worsened under stimulation, are detailed in Table 2. Fig. 1 displays the change in NMF2S scores between OFF-Dopa/OFF-Stim and OFF-Dopa/ON-Stim conditions at V2, illustrating the overall reduction in non-motor fluctuation severity under active stimulation. All DBS-related effects on NMF are expressed as percentage change relative to OFF-stimulation values in order to account for inter-individual variability in baseline NMF severity.

**Table 2:** NMF2S scores in the OFF-Dopa/OFF-Stim and OFF-Dopa/ON-Stim conditions.

NMF2S	Frequency OFF-Dopa/OFF-Stim (%)	Frequency OFF-Dopa/ON- Stim (%)	Severity OFF-Dopa/OFF-Stim (mean (SD))	Severity OFF-Dopa/ON- Stim (mean (SD))	Severity change OFF–ON (relative to Stim)*	Mean OFF/ONImprovement	Patients improved (%)	Patients unchanged (%)	Patients worsened (%)	Most disabling item OFF-Dopa/OFF-Stim (%)	Most disabling item OFF-Dopa/ON- Stim (%)
1. Anxiety	82,3%	64,6%	4,2 (2,9)	2,3 (2,5)	p < 0,001	45,4%	59,6%	28,5%	11,9%	12,6%	9,6%
2. Apathy	84,4%	71,8%	5,2 (3,5)	3,2 (3,1)	p < 0,001	38,9%	56,9%	29,0%	14,1%	8,0%	12,6%
3. Pain	66,8%	46,6%	3,2 (3,1)	1,8 (2,6)	p < 0,001	44,2%	47,4%	43,3%	9,3%	11,9%	11,3%
4. Fatigue	89,9%	79,5%	5,5 (3,0)	3,4 (2,9)	p < 0,001	37,4%	63,7%	23,3%	13,0%	36,0%	30,9%
5. Difficulty concentrating	87,0%	65,5%	4,6 (2,9)	2,5 (2,6)	p < 0,001	46,3%	67,2%	20,3%	12,5%	10,0%	10,9%
6. Sadness	59,2%	45,3%	2,5 (2,8)	1,4 (2,2)	p < 0,001	43,2%	43,2%	44,3%	12,5%	0,0%	0,0%
7. Dyspnea	47,3%	35,7%	1,8 (2,6)	1,1 (2,1)	p < 0,001	40,8%	31,1%	59,3%	9,6%	2,7%	3,5%
8. Restless legs	69,9%	54,3%	3,7 (3,4)	2,4 (3,0)	p < 0,001	35,5%	47,4%	37,0%	15,6%	14,6%	12,6%
9. Urinary disorders	43,5%	33,1%	1,6 (2,4)	1,1 (2,2)	p < 0,001	28,1%	23,0%	67,8%	9,3%	1,1%	2,6%
10. Irritability	50,5%	39,4%	2,1 (2,7)	1,3 (2,2)	p < 0,001	40,3%	35,6%	57,8%	6,7%	0,8%	2,2%
11. Excessive sweating	44,8%	33,8%	1,8 (2,6)	1,2 (2,3)	p < 0,001	33,1%	27,3%	62,7%	10,0%	2,3%	3,9%

SD: standard deviation

*Paired comparisons between OFF- and ON-stimulation were performed using the Wilcoxon signed-rank test. To account for multiple testing across the 11 NMF2S items, p-values were Bonferroni-corrected within this table (adjusted p-values reported; equivalent significance threshold α = 0.05/11 = 0.0045).

Among the 11 NMF symptoms studied, fatigue was the most frequent and severe, both with and without stimulation (reported by 89.9% after surgery; OFF Stim score: 5.5/10, ON Stim: 3.4/10). Urinary disorders, described as retention or urgency, were the least frequent and severe post-surgery (43.5%; OFF: 1.6/10, ON: 1.1/10). Dyspnea and excessive sweating were also less common and less severe.

All items improved significantly with DBS (p < 0.001, Wilcoxon test), with improvements ranging from 28.1% (urinary) to 46.3% (concentration difficulties).

Overall the NMF2S score improved by 41.1% in the ON Stim condition. Examining the different dimensions individually, DBS led to 44.8% improvement in psychiatric NMF, 43.5% in cognitive, and 38.0% in dysautonomic symptoms. The mean number of reported NMF dropped from 7.2 (OFF) to 5.6 (ON) items (p < 0.001).

In the ON Stim condition, 80.7% of patients improved their NMF2S score, with 5.9% of the population no longer reporting any NMF. When examining the different NMFs in detail, the proportion of patients who improved with stimulation ranges from 67.2% (concentration) to 23.0% (urinary disorders). Additionally, 5.2% showed no change, and 14.1% worsened.

Fatigue was most often cited as the most bothersome symptom at V2 (36.0% in OFF, 30.9% in ON). Urinary issues and irritability were rarely named as most annoying.

In OFF Stim, 41.2% rated their non-motor discomfort as worse than motor, versus 39.7% in ON. Conversely, 45.6% (OFF) and 43.3% (ON) rated motor discomfort as worse. The remaining patients were unsure.

### 3.3 Factors associated with NMFs one year after surgery

Table 3 shows the exploratory correlations between NMF2S scores in the OFF-Stim and ON-Stim conditions and the characteristics of our patients at V2.

**Table 3:** Relationships between clinical data and overall NMF2S total scores in the OFF-Stim and ON-Stim conditions.

Characteristics	NMF2S OFF-Dopa/OFF-Stim	NMF2S OFF-Dopa/ON-Stim
PDQ-39	0.410^**^	0.456^**^
Hoehn & Yahr OFF	0.183^**^	0.206^**^
Hoehn & Yahr ON	0.141*	**0.113**
Schwab & England (OFF)	−0.243^**^	−0.221^**^
Schwab & England (ON)	−0.223^**^	−0.019
MDS-UPDRS I	0.345^**^	0.349^**^
MDS-UPDRS II OFF	0.338^**^	0.247^**^
MDS-UPDRS II ON	0.262^**^	0.290^**^
MDS-UPDRS III OFF-Dopa/OFF-Stim	0.326^**^	0.092
MDS-UPDRS III OFF-Dopa/ON-Stim	0.141*	−0.002
MDS-UPDRS III modification by stimulation	0.02	0.078
MDS-UPDRS IV	0.210^**^	0.216^**^

Correlation coefficient Rho (Spearman), ρ. *: Correlation is significant at the 0.05 level (two-tailed); **: Correlation is significant at the 0.01 level (two-tailed).

Whether in the OFF-Dopa/OFF-Stim or OFF-Dopa/ON-Stim condition, higher NMF2S scores were significantly correlated with female gender, lower QoL, higher scores on MDS-UPDRS I, MDS-UPDRS II OFF, MDS-UPDRS II ON, MDS-UPDRS IV, and more severe motor score assessed by MDS-UPDRS III (Spearman correlations, p-values adjusted for multiple comparisons).

However, we found no correlation between the motor and the non-motor response to DBS (using the MDS-UPDRS III OFF-Dopa/ON-Stim scores and the NMF2S OFF-Dopa/ON-Stim scores).

### 3.4 Preoperative factors associated with the non-motor effect of DBS

For the 153 patients who participated in both pre-surgical [6] and post-surgical studies, we examined the pre-surgical factors associated with greater improvement of NMF2S due to DBS (Table 4).

**Table 4:** Relationship between the presurgical characteristics of patients and the non-motor effect of DBS at one-year post surgery.

Pre-surgical characteristics	Non-motor effect of DBS at one-year post surgery
Disease duration	0,068
Age	−0,036
PDQ-39	−0,019
Hoehn & Yahr OFF	0,076
Hoehn & Yahr ON	−0,023
Schwab & England (OFF)	−0,111
Schwab & England (ON)	0,014
MDS-UPDRS I	−0,008
MDS-UPDRS II OFF	0,031
MDS-UPDRS II ON	−0,003
MDS-UPDRS III OFF-Dopa	0,113
MDS-UPDRS III ON-Dopa	0,101
MDS-UPDRS III modification by levodopa	−0,036
MDS-UPDRS IV	−0,015
Non motor effect of levodopa	
NMF2S global score	0,119
NMF2S psychiatric score	0,043
NMF2S cognitive score	0,173†
NMF2S dysautonomic score	0,024

Spearman correlation coefficients (ρ).

p-values were adjusted for multiple comparisons using Bonferroni correction (m = 4; adjusted α≈0.0125).

Correlations that reached p < 0.05 before correction but did not remain significant after correction are marked with †.

We found no significant correlations between the non-motor effects of DBS (defined by the percentage of modification of NMF2S between OFF-Dopa/OFF-Stim and OFF-Dopa/ON-Stim conditions) and the presurgical characteristics of patients (disease duration of PD, age, PDQ39, Hoehn & Yahr scores, Schwab & England scores, and UPDRS I, II, III and IV). We also found that the presurgical motor response to levodopa (percentage change in UPDRS III with levodopa) was not correlated with the non-motor effects of DBS one year after surgery but was correlated with motor improvement following DBS (ρ = 0.39; p = 2.92 × 10^−11^). Moreover, even the pre-surgical non-motor effect of levodopa (assessed by NMF2S) [5] was not significantly correlated with the non-motor effect of DBS at one-year (ρ = 0.119 and p = 0.161).

By analyzing the dimension scores of our scale, we found that the pre-surgical effect of levodopa on the cognitive dimension of NMF2S was significantly correlated with the global non-motor effect of DBS after one year (ρ = 0.173 and p = 0.043) (Table 4).

### 3.5 Post-surgical factors related to the non-motor effect of DBS

In the post-surgical context, we found a significant correlation between DBS-related non-motor improvement and a better QoL of patients (ρ = − 0.163; p < 0.001). We also found no association between the magnitude of motor improvement induced by DBS (assessed as the percentage change in MDS-UPDRS III between OFF-Dopa/OFF-Stim and OFF-Dopa/ON-Stim conditions) and the corresponding non-motor effect of DBS (ρ =  − 0.041; p = 0.506).

## 4 Discussion

This study evaluated the impact of STN-DBS using a new real-time method to assess NMF in Parkinson’s disease and factors associated with non motor fluctuations. One year after surgery, DBS decreased the severity and frequency of all NMF but remained less effective than levodopa, especially for psychiatric fluctuations. No correlation was found between motor and non-motor improvements, and no preoperative clinical factor showed a robust association with post-DBS NMF outcomes. These findings underscore the need for a deeper understanding of non-motor responses to DBS and their implications for patient selection.

### 4.1 Effectiveness of deep brain stimulation on fluctuation of non-motor symptoms

Our results confirm that STN-DBS significantly improves NMF in Parkinson’s disease. Using the NMF2S scale, we observed a 41.1% reduction in overall NMF severity one year after surgery. The most improved symptoms were anxiety, concentration difficulties, and pain, whereas urinary disorders showed the least responsiveness.

These findings align with Witjas et al. [5] and Borgohain et al. [3], which showed that DBS modulates NMS, including psychiatric and autonomic dysfunctions. In contrast, Witjas et al. [5] reported greater improvement in pain and cognitive NMF than in psychiatric domains, possibly due to their retrospective evaluation method.

Our study adds new evidence by showing that NMF improvements persist one year after surgery in a large cohort, using an immediate-response scale rather than retrospective recall. Unlike traditional retrospective tools (e.g., NoMoFa [13], UPDRS [14]), the NMF2S allows real-time assessment, reducing recall bias and providing an intuitive visual index.

Its ability to quantify symptom severity at a specific moment makes it a valuable tool for evaluating treatment efficacy and monitoring NMF longitudinally. However, while immediacy enhances reliability, it also limits long-term assessment and may increase sensitivity to placebo. Our results also confirm that NMF severity is higher in female patients, consistent with recent studies [[15], [16]]. This gender difference, also observed in early PD, highlights the need for sex-specific approaches and closer attention to NMF in women when adjusting dopaminergic treatment.

### 4.2 Lack of correlation between motor and non-motor fluctuation improvements under DBS

Our analysis identified no significant correlation between motor and non-motor symptom improvements under DBS. Although DBS reduced MDS-UPDRS-III motor scores by 58.2%, this improvement was not associated with the NMF response.

This dissociation is consistent with Witjas et al. [5], who first demonstrated that motor and non-motor fluctuations respond independently to DBS. Ortega-Cubero et al. [17] similarly reported that DBS reduced the severity of non-motor fluctuations without affecting their frequency. Ledda et al. [13] also found no correlation between the motor and non-motor responses to DBS, but observed a strong association between the improvement of non-motor fluctuations and the reduction of motor complications (including dyskinesias, OFF periods, and dystonia).

Altogether, these findings indicate that DBS modulates distinct neural circuits underlying motor and non-motor domains. This relative independence supports the development of more targeted neuromodulation strategies, including alternative stimulation sites such as the GPi or limbic subregions of the STN, which may better address non-motor symptoms.

In line with previous reports, NMF severity was significantly associated with poorer quality of life, as measured by the PDQ-39 [[4], [18], [19]]. Rather than reiterating this well-established relationship, the present findings highlight the clinical implications of the dissociation between motor and non-motor responses to DBS.

Jost et al. [16] observed that improvement in patients' QoL at 36 months after surgery is more strongly influenced by NMS than by the motor response to DBS, particularly difficulties in experiencing pleasure and challenges in maintaining concentration. These findings may help explain why some patients report dissatisfaction with DBS, even when clinicians note substantial motor improvements [[20], [21]].

### 4.3 DBS provides less improvement in NMF compared to levodopa

While DBS improved all NMF domains, its effect remained smaller than levodopa across psychiatric, cognitive, and dysautonomic symptoms.

In the same population (PREDISTIM cohort), Faggianelli et al. [6] reported that levodopa administration during the levodopa challenge yielded a 56.5% preoperative reduction in NMF severity, whereas in our study DBS achieved a 41.1% reduction one year postoperatively. This difference was most pronounced in the psychiatric domain, where levodopa achieved a 62.2% improvement, whereas DBS reached only 44.8%**.**

One possible explanation is that levodopa directly enhances dopaminergic neurotransmission, whereas DBS modulates neural circuits indirectly. Previous studies [22] support this hypothesis, suggesting that levodopa exerts broad effects across multiple dopaminergic pathways**,** whereas DBS primarily modulates a specific brain network. Another potential explanation relates to the spatial specificity of STN stimulation. In routine clinical practice, electrodes are primarily targeted to the dorsolateral sensorimotor STN to optimize motor benefit, whereas non-motor symptoms are thought to involve associative and limbic subregions. Variability in electrode positioning and volume of tissue activated may therefore contribute to the more modest and heterogeneous DBS effects observed on non-motor fluctuations and warrants investigation in future studies.

Building on this anatomical distinction, psychiatric symptoms such as anxiety and apathy are strongly influenced by limbic dopaminergic pathways, particularly involving the ventral tegmental area (VTA), which may be less effectively modulated by standard STN stimulation [[23], [24]]. Some authors have suggested that alternative DBS targets, like the Globus Pallidus Internus (GPi), may be more effective for psychiatric NMS. Indeed, El Ghazal et al. [25] found in a *meta*-analysis that GPi-DBS produced superior effects on depression and anxiety, leading to an improved quality of life, even though it provided less relief from motor symptoms. According to them, the GPi may be a superior DBS target for non-motor symptoms due to its larger, more segregated motor territory, reducing unintended stimulation of limbic circuits. In contrast, the STN’s proximity to affective pathways increases the risk of mood disturbances, explaining the greater improvement in depression and UPDRS-I scores seen with GPi stimulation.

A noteworthy finding is that fatigue remained the most persistent non-motor symptom post-DBS, even though 63.7% of patients reported some improvement with stimulation, compared to 73.9% who experienced relief with levodopa before surgery [6]. This raises concerns regarding whether fatigue should truly be classified as a fluctuating symptom. Studies have suggested that post-DBS fatigue may be linked to preoperative depression [26], indicating that this symptom should be considered prior to surgery. Given the substantial impact of fatigue on quality of life, patients should be systematically screened for depression prior to surgery, and appropriate treatment should be provided when necessary.

### 4.4 Preoperative factors associated with NMF improvement

Our study further demonstrated that no preoperative clinical variable reliably showed a significant association with NMF improvement following DBS when considering correction for multiple comparisons. Neither disease duration, nor age, nor preoperative symptom severity showed a significant correlation with post-DBS NMF outcomes.

Notably, although it has a well-established predictive value for motor outcomes, preoperative levodopa responsiveness in NMF was not significantly associated the NMF response to DBS. This study is the first to explore the association between preoperative levodopa responsiveness in the non-motor domain and post-DBS NMF outcomes.

The only preoperative factor significantly associated with post-DBS NMF improvement was the levodopa response in the cognitive domain. Although the association did not remain statistically significant after Bonferroni correction for multiple comparisons, the observed trend suggests a potential relationship that should be investigated in larger, prospectively designed cohorts with adequate statistical power and pre-specified hypotheses.

Hamani et al. [27] described the diverse functions of the STN through its projections, highlighting its role in cognition via connections to the limbic circuit, including limbic cortical afferents such as the orbitofrontal cortex and anterior cingulate gyrus.

In addition, Prell [28] reported an association between non-motor symptoms (NMS) and hypometabolism of the anterior cingulate cortex, a region well known for its role in cognition.

Building on this evidence, our findings suggest that preoperative levodopa responsiveness in the cognitive domain may be associated with overall NMF improvement after DBS. This could indicate that the degree of non-motor symptom responsiveness to DBS is dependent on the integrity of frontostriatal and limbic dopaminergic circuits [[29], [30]].

Future research should further investigate the complex interaction between cognition and NMF, as these domains may share overlapping neural mechanisms. The identification of neuroimaging biomarkers reflecting frontostriatal integrity or cortical dysfunction could provide valuable insights, potentially refining patient selection and optimizing DBS surgical targets.

These findings highlight the complexity of NMF modulation under DBS and suggest that, beyond the levodopa challenge, alternative approaches should be explored to predict the DBS outcomes on NMF. They also emphasize the need for more reliable predictive biomarkers.

### 4.5 Limitations

This study has limitations.

The retrospective and ancillary nature of this analysis resulted in heterogeneity and variable availability of some collected data, including preoperative NMF assessments and detailed dopaminergic medication subclasses.

As a result, the analyses were not designed to support confirmatory or fully adjusted predictive modeling, and the present findings should be interpreted as exploratory associations rather than independent predictors.

Electrode localization and active contact position were not available in a standardized manner across centers, precluding a formal analysis of the impact of stimulation site within the STN on non-motor outcomes.

In addition, non-motor fluctuations were assessed under standardized OFF- and ON-stimulation conditions, providing an immediate and memory-independent evaluation of DBS-related effects, but this approach does not capture the full temporal variability of non-motor fluctuations experienced in daily life.

## 5 Conclusion

This multicentric study confirms that STN-DBS effectively alleviates NMS and improves non-motor fluctuations in Parkinson’s disease, with effects sustained after one year. Preoperative dopaminergic responsiveness in the cognitive domain is associated with postoperative DBS benefit and supports the relative independence of motor and non-motor outcomes.

Beyond confirming prior reports, our findings show that NMF improvement occurs independently of motor effects, and that preoperative cognitive dopaminergic responsiveness shows a trend toward an association with post-DBS benefit. Unlike motor fluctuations, NMFs display a less predictable response to DBS, and their improvement is not associated with motor relief. No other reliable preoperative clinical factors showed significant associations with post-DBS NMF outcomes.

Given the strong association between NMS and quality of life, these results highlight the need for a deeper understanding of NMF pathophysiology and for more individualized approaches to DBS patient selection.

## CRediT authorship contribution statement

**Christian Matta:** Writing – original draft, Methodology, Data curation. **Camille Comet:** Writing – original draft, Investigation, Data curation. **Corentin Gaillard:** Writing – review & editing. **Florent Faggianelli:** Writing – review & editing, Data curation. **Stephan Grimaldi:** Writing – review & editing, Investigation. **Tatiana Witjas:** Writing – review & editing, Methodology, Investigation. **Solène Ansquer:** Investigation. **Cécile Hubsch:** Investigation, Writing – review & editing. **Mathieu Anheim:** Investigation. **Caroline Moreau:** Investigation. **Elodie Hainque:** Investigation. **Sophie Drapier:** Investigation. **Béchir Jarraya:** Investigation. **Chloé Laurencin:** Investigation. **Dominique Guehl:** Investigation. **Lucie Hopes:** Investigation. **Christine Brefel-Courbon:** Investigation. **Melissa Tir:** Investigation. **Ana Marques:** Investigation. **Tiphaine Rouaud:** Investigation. **David Maltete:** Investigation. **Caroline Giordana:** Investigation. **Karine Baumstarck:** Methodology. **Olivier Rascol:** Investigation. **Jean-Christophe Corvol:** Investigation. **Fabienne Ory-Magne:** Investigation. **Anne-Sophie Rolland:** Investigation. **David Devos:** Investigation. **J-P Azulay:** Writing – review & editing, Investigation, Conceptualization. **Alexandre Eusebio:** Writing – review & editing, Supervision, Investigation, Conceptualization.

## Declaration of competing interest

The authors declare that they have no known competing financial interests or personal relationships that could have appeared to influence the work reported in this paper.

## References

[1] Witjas T., Kaphan E., Azulay J.P. (2002). Nonmotor fluctuations in PD. Neurology.

[2] Aissi M., Douma R., Narjas M., Rrafik M., Younes S., Frih A.M. (2016). Fluctuations non motrices au cours de la maladie de Parkinson. Rev. Neurol..

[3] Borgohain R., Kandadai R.M., Jabeen A., Kannikannan M.A. (2012). Nonmotor outcomes in Parkinson’s disease: is deep brain stimulation better than dopamine replacement therapy?. Ther. Adv. Neurol. Disord..

[4] Georgiev D., Mencinger M., Rajnar R., Mušič P., Benedičič M., Flisar D. (2021). Long-term effect of STN-DBS on non-motor symptoms: a four-year prospective study. Parkinsonism Relat. Disord..

[5] Witjas T., Kaphan E., Régis J., Jouve E., Chérif A.A. (2007). Effects of chronic STN stimulation on NMFs. Mov. Disord..

[6] Faggianelli F., Witjas T., Azulay J.P., Benatru I., Hubsch C., Anheim M. (2024). ON/OFF non-motor evaluation in Parkinson’s disease. J. Neurol. Neurosurg. Psychiatry.

[7] Cavallieri F., Fraix V., Bove F., Mulas D., Tondelli M., Castrioto A. (2021). Predictors of long-term outcome of subthalamic stimulation in Parkinson disease. Ann. Neurol..

[8] Hughes A.J., Daniel S.E., Kilford L. (1992). Accuracy of clinical diagnosis of idiopathic Parkinson’s disease. J. Neurol. Neurosurg. Psychiatry.

[9] Jenkinson C., Fitzpatrick R., Peto V., Greenhall R., Hyman N. (1997). The PDQ-39: development and validation. Age Ageing.

[10] Auquier P., Sapin C., Ziegler M., Tison F., Destée A., Dubois B. (2002). Validation of the French version of the Parkinson’s Disease Questionnaire (PDQ-39). Rev. Neurol. (Paris).

[11] Chaudhuri K.R., Schrag A., Weintraub D., Rizos A., Rodriguez-Blazquez C., Mamikonyan E. (2020). The Movement Disorder Society Nonmotor Rating Scale: initial Validation Study. Mov. Disord..

[12] Kleiner G., Fernandez H.H., Chou K.L., Fasano A., Duque K.R., Hengartner D. (2021). Validation of the NoMoFa questionnaire. Mov. Disord..

[13] Ledda C., Imbalzano G., Tangari M.M., Covolo A., Donetto F., Montanaro E. (2024). NoMoFa for DBS-related NMFs. Parkinsonism Relat. Disord..

[14] Jafari N., Pahwa R., Nazzaro J.M., Arnold P.M., Lyons K.E. (2016). MDS-UPDRS to assess non-motor symptoms after STN DBS. Int. J. Neurosci..

[15] Donzuso G., Cicero C.E., Vinciguerra E., Sergi R., Luca A., Mostile G. (2023). Gender differences in non-motor fluctuations in Parkinson’s disease. J. Neural Transm..

[16] Kang K.W., Choi S.M., Kim B.C. (2022). Gender Differences in Early PD. Medicine.

[17] Ortega-Cubero S., Clavero P., Irurzun C. (2013). Effect of STN-DBS on NMFs: 2-year follow-up. Parkinsonism Relat. Disord..

[18] Jost S.T., Visser-Vandewalle V., Rizos A. (2021). Non-motor predictors of QoL after STN-DBS. NPJ Parkinsons Dis..

[19] Nazzaro J.M., Pahwa R., Lyons K.E. (2011). Impact of STN-DBS on non-motor symptoms. Parkinsonism Relat. Disord..

[20] Maier F., Lewis C.J., Horstkoetter N., Eggers C., Kalbe E., Maarouf M. (2013). Expectations and perceived outcome of DBS. J. Neurol. Neurosurg. Psychiatry.

[21] Schüpbach M., Gargiulo M., Welter M.L., Mallet L., Béhar C., Houeto J.L. (2006). Neurosurgery in PD: mind–body dissociation. Neurology.

[22] Muthuraman M., Koirala N., Ciolac D. (2018). DBS and L-DOPA: mechanisms and applications. Front. Neurol..

[23] Krack P., Ardouin C., Funkiewiez A. (2001). Influence of STN stimulation on limbic loop. In: Basal Ganglia and Thalamus..

[24] Kim H.J., Jeon B.S., Paek S.H. (2015). Nonmotor symptoms and STN-DBS. J Mov Disord..

[25] El Ghazal N., Nakanishi H., Martinez-Nunez A.E., Al Sabbakh N.K., Segun-Omosehin O. (2023). Effects of deep brain stimulation on mood. Cureus.

[26] Magalhães A.D., Amstutz D., Petermann K. (2024). Acute psychotropic effects of STN stimulation. BMJ Neurol Open..

[27] Hamani C., Saint-Cyr J.A., Fraser J., Kaplitt M., Lozano A.M. (2004). The subthalamic nucleus in movement disorders. Brain.

[28] Prell T. (2018). Structural and functional brain patterns of NMS in PD. Front. Neurol..

[29] Cools R., Barker R.A., Sahakian B.J., Robbins T.W. (2001). Cognitive function. Cereb. Cortex.

[30] Monchi O., Petrides M., Mejia-Constain B., Strafella A.P. (2007). Executive processing in PD. Brain.

