# Supplementary material for: Effects of deep brain stimulation on non motor fluctuations in Parkinson’s disease (assessed with the NMF severity scale)
Source: Clin Park Relat Disord. 2026 Feb 8;14:100426. doi: 10.1016/j.prdoa.2026.100426 (PMC12915265; doi:10.1016/j.prdoa.2026.100426)
Supplement: Supplementary Data 1 [file mmc1.docx]

**Supplementary Materials**

Statistical analyses were conducted to evaluate the effect of STN-DBS on NMF and to identify potential predictors of improvement. All analyses were performed using SPSS 20.0, M-PLUS, and R (packages ltm, psych, and TAM). A significance threshold of p < 0.05 was applied to all statistical tests. Missing data were excluded from the analysis.

**2.3.1. Comparison of Non-Motor Fluctuations Before and After DBS**

The effect of STN-DBS on NMF was assessed by comparing NMF2S scores between OFF-Dopa/OFF-Stim and OFF-Dopa/ON-Stim conditions, one year after surgery. Wilcoxon signed-rank tests were used to analyze within-subject differences between these two conditions. The analysis was conducted for the global NMF2S score as well as its three specific dimensions: psychiatric, cognitive, and dysautonomic.

**2.3.2. Analysis of DBS Effects on Specific NMF Domains**

To further assess the impact of DBS, the variation in each individual NMF2S symptom (anxiety, apathy, fatigue, pain, urinary dysfunction, irritability, etc.) was analyzed. For each symptom, the percentage of patients reporting it and the mean change in symptom severity between OFF-Stim and ON-Stim conditions were calculated. The Wilcoxon signed-rank test was used to evaluate statistical significance for each symptom.

**2.3.3. Correlation Between NMF Severity and Clinical Characteristics**

Spearman’s correlation coefficients, Rho (ρ) were used to examine associations between NMF severity (NMF2S scores in OFF-Stim and ON-Stim conditions) and various clinical variables. The explored parameters included Hoehn & Yahr stage, MDS-UPDRS scores, quality of life (PDQ-39), and Schwab & England scores. This analysis aimed to determine whether certain clinical markers were associated with greater NMF severity before and after DBS.

**2.3.4. Relationship Between Motor Improvement and NMF Improvement**

To assess whether motor and non-motor responses to DBS were correlated, Spearman’s correlation tests were used to compare the variation in MDS-UPDRS III scores (OFF-Dopa/OFF-Stim → OFF-Dopa/ON-Stim) with changes in NMF2S scores under the same conditions. This analysis aimed to determine whether patients with better motor improvement under DBS also exhibited greater NMF improvement.

**2.3.5. Identification of Predictors of NMF Improvement After DBS**

To identify preoperative predictors of NMF improvement after one year of DBS, correlations between preoperative patient characteristics and NMF2S score variation were analyzed. The tested variables included age, disease duration, MDS-UPDRS I-IV scores, preoperative motor response to L-Dopa (change in MDS-UPDRS III score under L-Dopa), and preoperative non-motor response to L-Dopa (change in NMF2S score under L-Dopa). Spearman’s correlation analyses were used to test these associations.
